# Swept-source optical coherence tomography angiography findings in a case of primary vitreoretinal lymphoma over a three-year follow-up

**DOI:** 10.1186/s12886-024-03438-1

**Published:** 2024-04-25

**Authors:** Emilia Maggio, Francesco Bauci, Antonio Polito, Fabrizio Arena, Grazia Pertile

**Affiliations:** IRCCS Sacro Cuore Don Calabria Hospital, Via Don Sempreboni 5, Negrar, Verona 37024 Italy

**Keywords:** Primary intraocular lymphoma, Vitreoretinal lymphoma, OCT-angiography, Chorioretinal biopsy, Ocular oncology

## Abstract

**Background:**

Vitreoretinal lymphoma (VRL) still represents a diagnostic challenge for retinal specialists. Early diagnosis and treatment are critical for a better prognosis. Several diagnostic tools have proven helpful in the identification of VRL abnormalities. However, swept-source OCT angiography (SS-OCT-A) findings and their long-term follow-up are yet to be explored.

**Case presentation:**

a 42-year-old man presented with blurred vision in his left eye for 2 weeks. He denied any systemic symptoms. A multimodal imaging examination was performed, raising the clinical suspicion of VRL and guiding the ensuing diagnostic procedures. The patient underwent treatment and at the last FU visit three years later, no disease signs were present on fundus examination, nor on oncologic evaluation. Some novel SS-OCT-A features were identified, and uncommonly reported findings were examined over a long-term follow-up. At baseline multiple hyperreflective alterations were detected on the enface outer retina slabs and choriocapillary analysis revealed low reflectance areas in the foveal and parafoveal areas. One month after the first presentation, multiple hyperreflective retinal lesions in a vertical shape were detected on OCT which appeared on midretinal slabs of enface SS-OCT-A as hyperreflective spots mainly located near second-order retinal vessels. These alterations remarkably reduced after treatment.

**Conclusion:**

SS-OCT-A may be a useful imaging technique in the detection of VRL, providing ophthalmologists additional findings that assist the diagnosis and follow-up of this disease. This may prove useful for a more timely and precise diagnosis, prompt therapy, and treatment response monitoring. The original aspects found in this case may provide grounds for future studies, ultimately fostering a better understanding of the disease.

## Background

Vitreoretinal lymphoma (VRL) is a rare intraocular neoplasm, representing < 1% of all intraocular tumors. Nevertheless, it is the most common intraocular lymphoproliferative pathology [1, 2]. It is considered a subset of primary central nervous system lymphoma (PCNSL) with ocular involvement on presentation [1–3]. Central nervous system (CNS) localization is very common, ranging from 35 to 90% of all cases throughout its course, as well as a typically asymmetric bilateral presentation. Women are more prone to develop this pathology, with presentation usually between the 4th and 6th decade of life [1–4].

VRL is often referred to as a masquerade syndrome for its uveitis-mimicking clinical features. Patients generally refer to an ophthalmologist complaining of blurred vision, floaters, or both. Presentation can be very similar to ocular inflammation with signs of iritis, vitritis, and retinitis. Due to this ambiguous appearance, VRLs may be misinterpreted and treated as uveitis, usually showing clinical improvement after steroid-based therapy. Recrudescence can be common with steroid tapering or discontinuation, and patients may undergo further steroid therapy cycles. This behavior can hamper physician judgement and lead to a significant delay in the diagnosis. The average time between symptom onset and histopathological diagnosis is reported to be 13.9 months [5]. However, early diagnosis and prompt treatment are critical for a better prognosis.

Several diagnostic tools have proven helpful to identify VRL abnormalities and thus assist diagnosis. However, OCT angiography (OCT-A) findings need further exploration, since little has been described to date [6–7], and long-term follow-up is still lacking. Enhancing knowledge in this field would facilitate early diagnosis and therapy response monitoring.

Herein, we report a case of VRL with the identification of novel swept-source OCT angiography (SS-OCT-A) features and the long-term analysis of uncommonly reported OCT-A findings.

## Case presentation

**Fig. 1:**
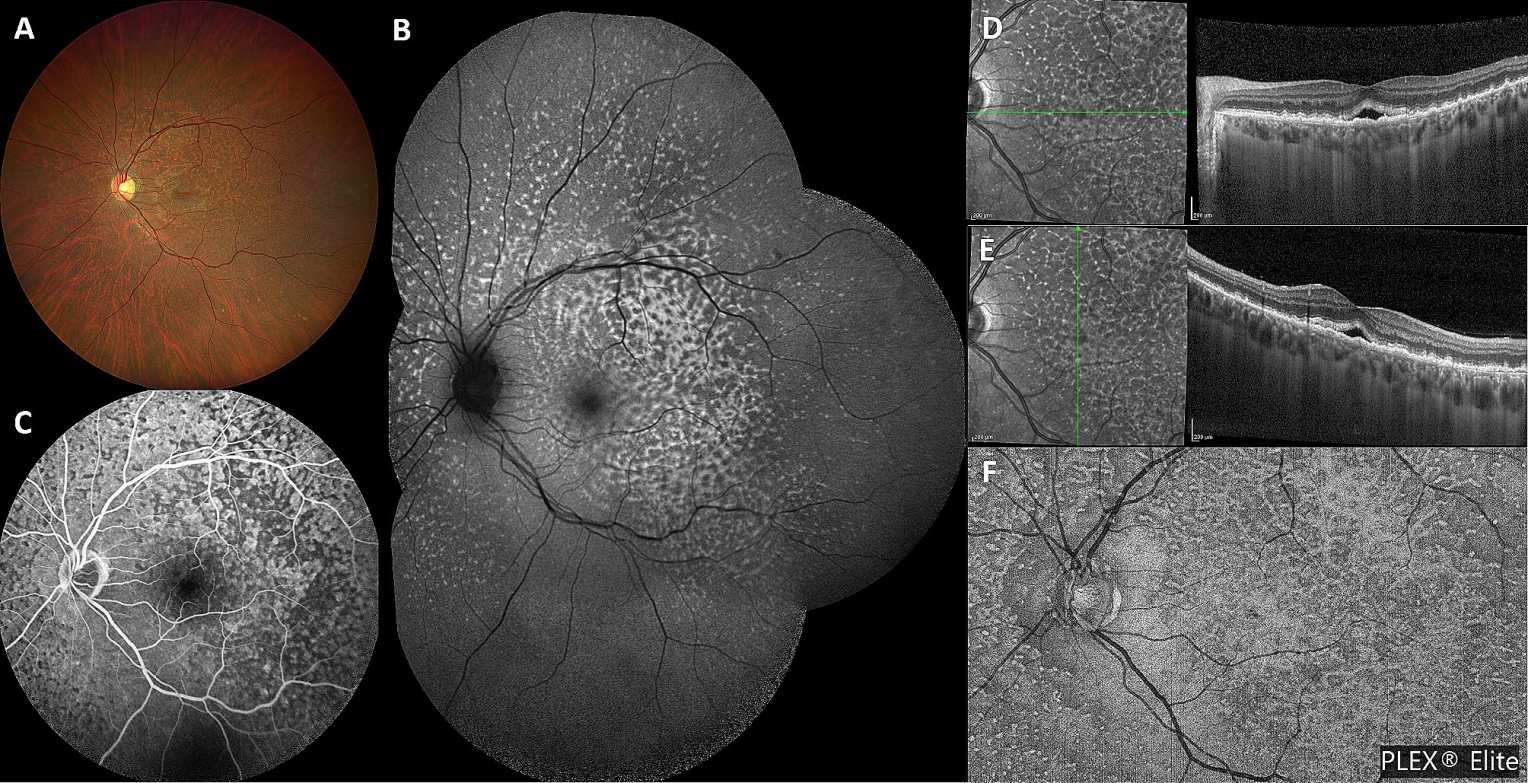
Multimodal imaging at first presentation. Baseline color fundus photo (**A**) showing multiple yellowish white deposits at the posterior pole and mid-periphery, appearing hyperautofluorescent on autofluorescence (**B**), and hypofluorescent on fluorescein angiography (**C**). (**D**, **E**) Baseline OCT scans showing multiple subretinal hyper-reflective infiltrates and a slight subfoveal neuroretinal detachment. (**F**) Sub-RPE deposits appearing hyperreflective on enface SS-OCT-A outer retina slabs, with a leopard spot pattern

**Fig. 2:**
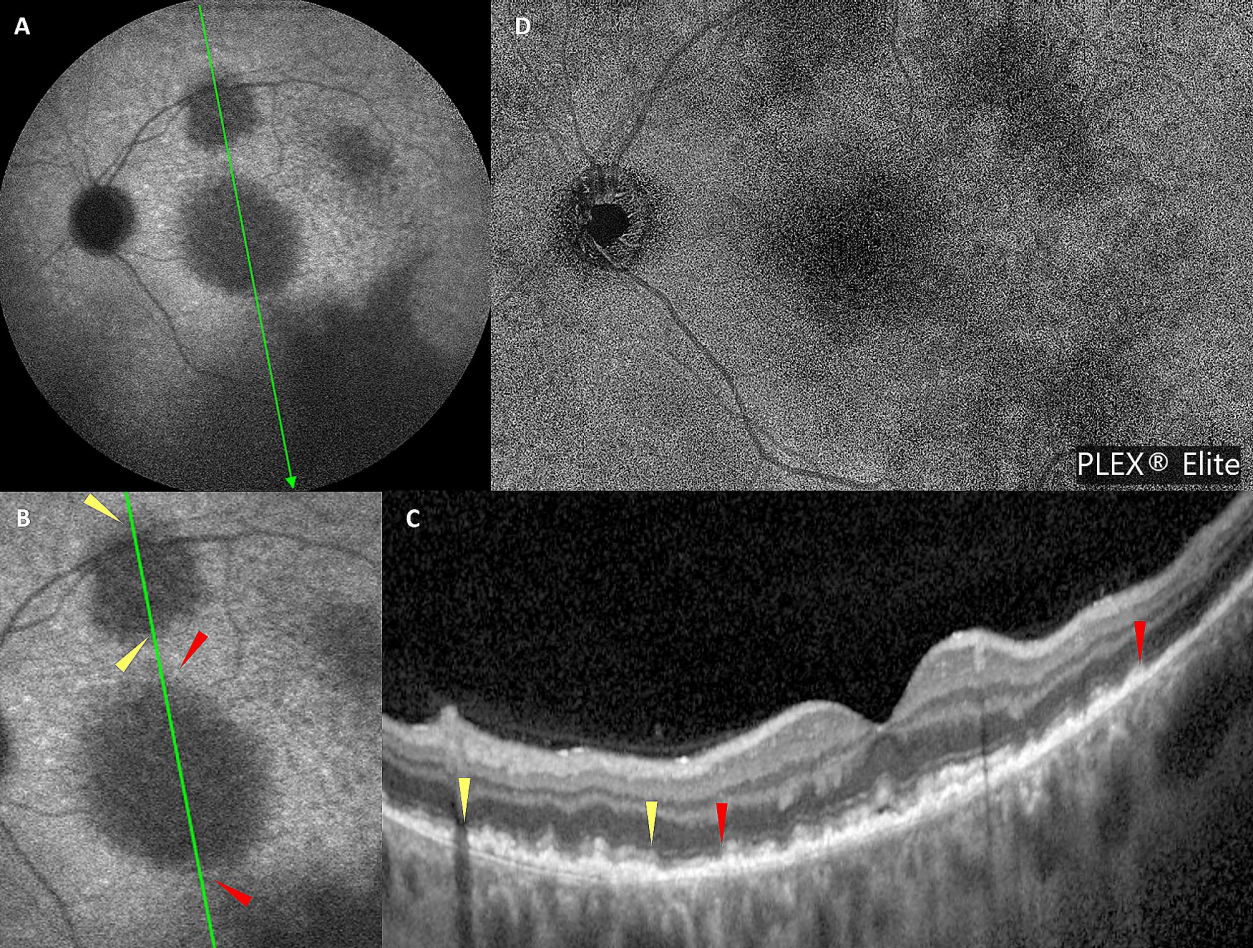
(**A**) Baseline late-phase ICGA angiograms revealing circumscribed hypofluorescent areas at the posterior pole, corresponding to sites with the most OCT alterations. (**B**) Magnification of above image highlighting hypofluorescent areas (yellow and red arrowheads) and corresponding OCT alteration (**C**). (**D**) SS-OCT-A choriocapillary analysis showing circumscribed low reflectance areas in foveal and parafoveal areas, corresponding to the hypofluorescent areas seen on ICGA

A 42-year-old white male presented with blurred vision in his left eye (LE) for 2 weeks and without systemic symptoms. On examination, best-corrected visual acuity (BCVA) was 20/20 in both eyes. Intraocular pressure was 17 mmHg OU. Clinical examination of the right eye (RE) was unremarkable. Slit lamp examination of the LE did not show any anterior segment abnormalities. Dilated fundoscopy of the LE showed a clear vitreous with multiple retinal yellowish white deposits at the posterior pole and mid-periphery (Fig. 1A). Optical coherence tomography (OCT) (Spectralis Heidelberg Engineering, Heidelberg, Germany) examination showed multiple hyper-reflective deposits under the retinal pigment epitelium (RPE) and a slight subfoveal neuroretinal detachment (SND) (Fig. 1D, E). On autofluorescence (AF) and fluorescein angiography (FA), the multifocal sub-RPE deposits appeared hyperautofluorescent and hypofluorescent, respectively, with no leakage on the late-phase angiograms (Fig. 1B, C). Indocyanine green angiography (ICGA) late angiograms revealed circumscribed hypofluorescent areas, corresponding to the sites with the most OCT alterations (Fig. 2A, B). Through swept-source OCT angiography, after projection removal, (SS-OCT-A, PLEX Elite 9000, Carl Zeiss Meditech Inc, Dublin, CA), multiple hyperreflective alterations were detected on the enface outer retina slabs (Fig. 1F), corresponding to the alterations identified through fundoscopy, OCT, AF, and FA. Upon SS-OCT-A analysis, the superficial and deep plexuses reconstructions showed no alterations of the retinal vascular network. On the contrary, choriocapillary analysis revealed circumscribed low reflectance areas in the foveal and parafoveal areas (Figs. 2C and 5A), which colocalized with the hypofluorescent areas seen in the late-phase ICGA angiograms (Fig. 2A).

**Fig. 3:**
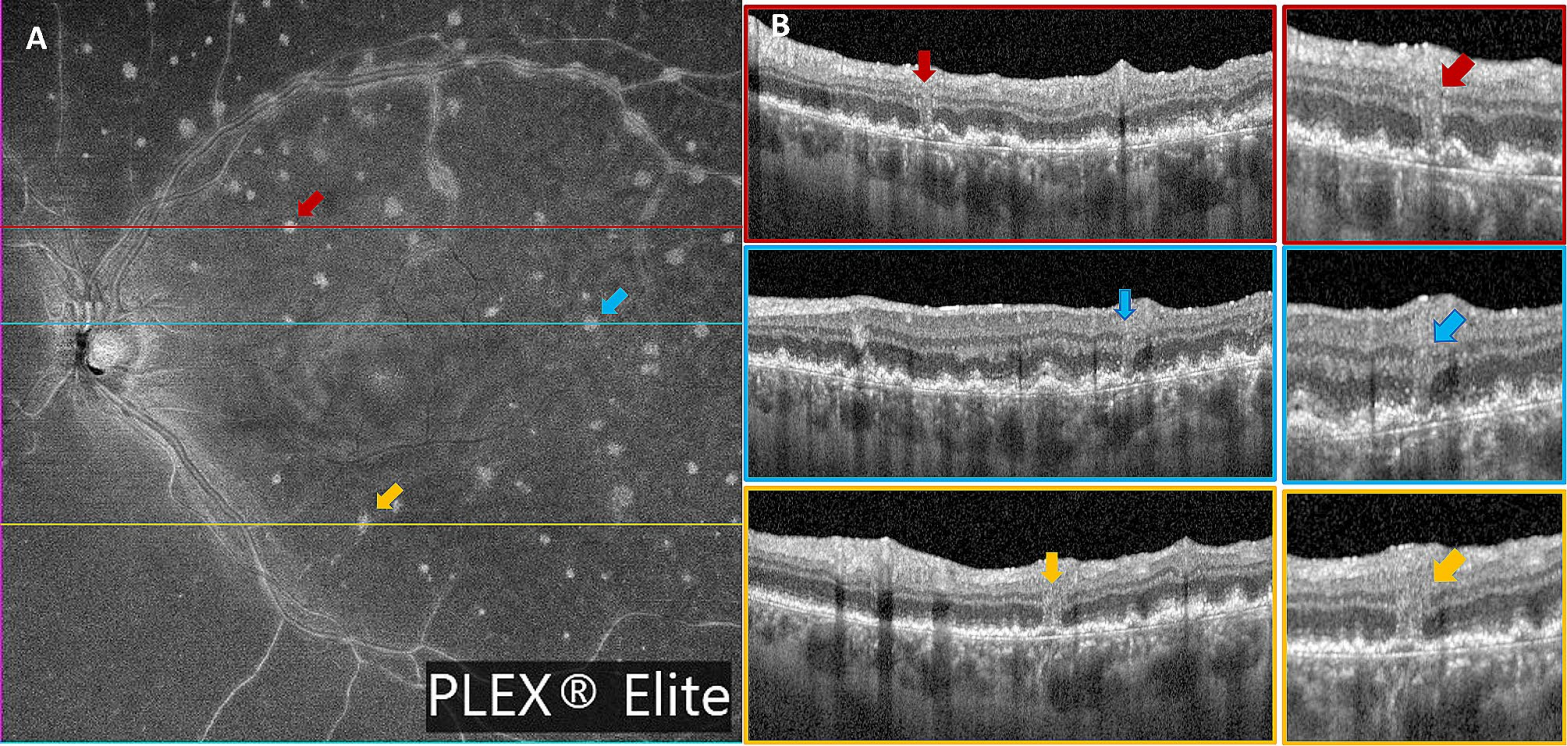
(A) Midretinal slab of enface SS-OCT-A, showing hyperreflective spots mainly located near second-order retinal vessels, corresponding to hyperreflective vertical retinal lesions. (B) OCT scans crossing the hyperreflective vertical retinal lesions

An extensive workup to rule out infectious posterior uveitis and systemic diseases was performed, including serologies for syphilis, toxoplasmosis, and tuberculosis, as well as screening for liver and renal function, glycemia, homocysteine levels, and inflammatory indexes. The results of all these examinations were negative. Cerebral magnetic resonance imaging (MRI) was performed and showed no abnormalities.

Based on the clinical presentation and multimodal imaging, a diagnosis of vitreretinal lymphoma was suspected. For confirmation, a diagnostic 27-gauge pars plana vitrectomy (PPV) was performed to obtain a vitreous sample for cytopathology. However, the results of this analysis were also negative.

Interleukin 10/Interleukin 6 (IL-10:IL6) ratio analysis on a vitreous sample was considered but was not available in our laboratory nor in neighboring hospitals. An MYD88 gene sequencing analysis of the aqueous humor, vitreous samples, and vitrectomy cassette fluid was performed, again with negative results. Consequently, a chorioretinal biopsy was planned.

Meanwhile, the patient complained of worsening LE visual acuity. Multimodal imaging showed a progression of the lesions in the LE and new involvement of the RE. One month after the first presentation, multiple new hyperreflective retinal lesions in a vertical shape were detected on OCT in the LE (Fig. 3B). On midretinal slabs of enface SS-OCT-A, these appeared as hyperreflective spots mainly located near second-order retinal vessels (Fig. 3A). These lesions were not detectable on baseline examinations and appeared like those previously referred to as perivascular flower-bud-like-lesions (PFBLs) [6].

**Fig. 4:**
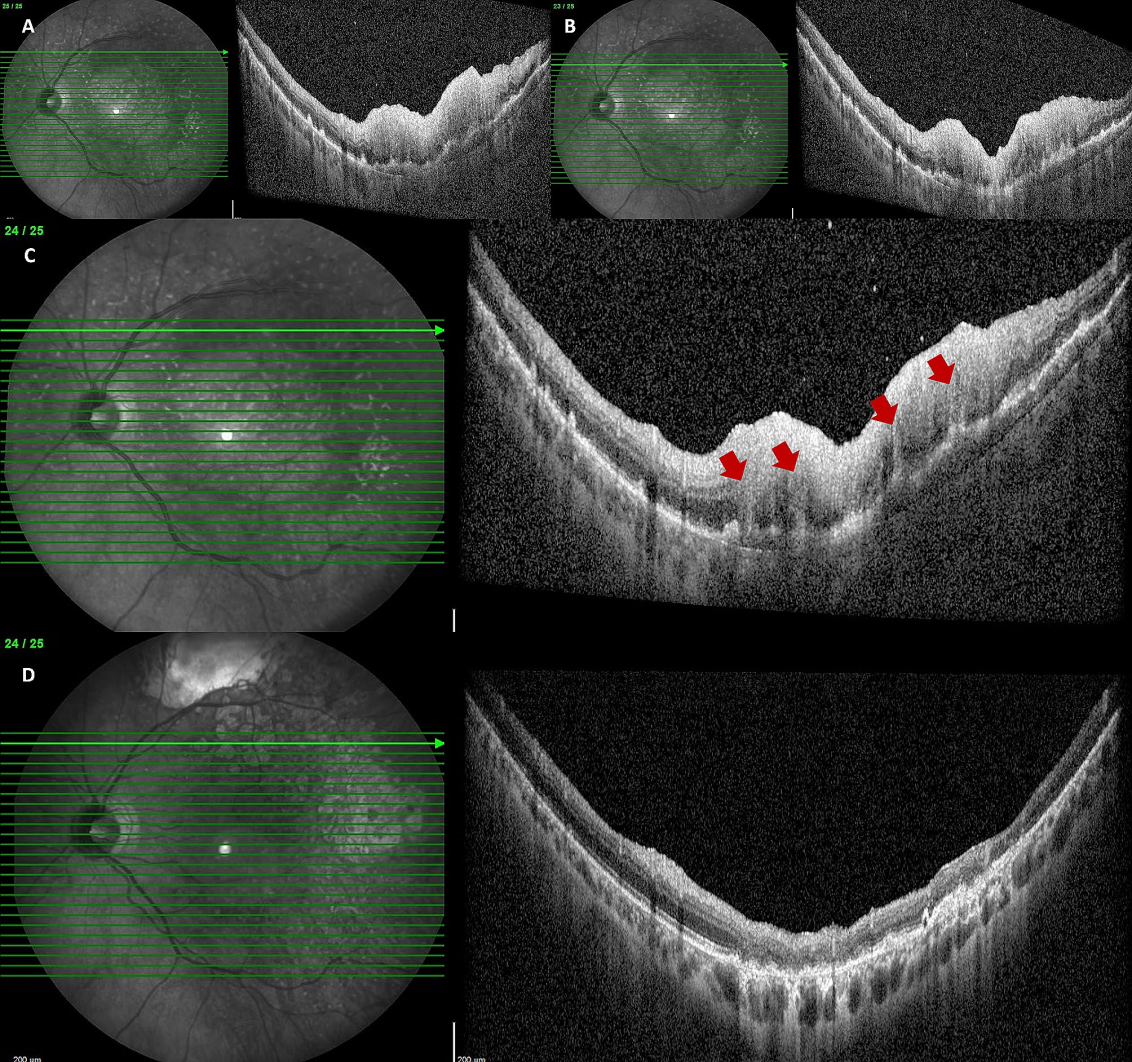
OCT scans in the superotemporal quadrant showing a large flat PED, along with an increase in the thickness and reflectivity of the overlying retina (**A**-**C**). Red arrows highlight vertical shaped retinal lesions, which appeared in continuity with the thick and hyperreflective overlying retina (**C**). (**D**) After chemotherapy and autologous stem cell transplant, the superior temporal flat PED regressed, along with a thickening of the overlying retina

**Fig. 5:**
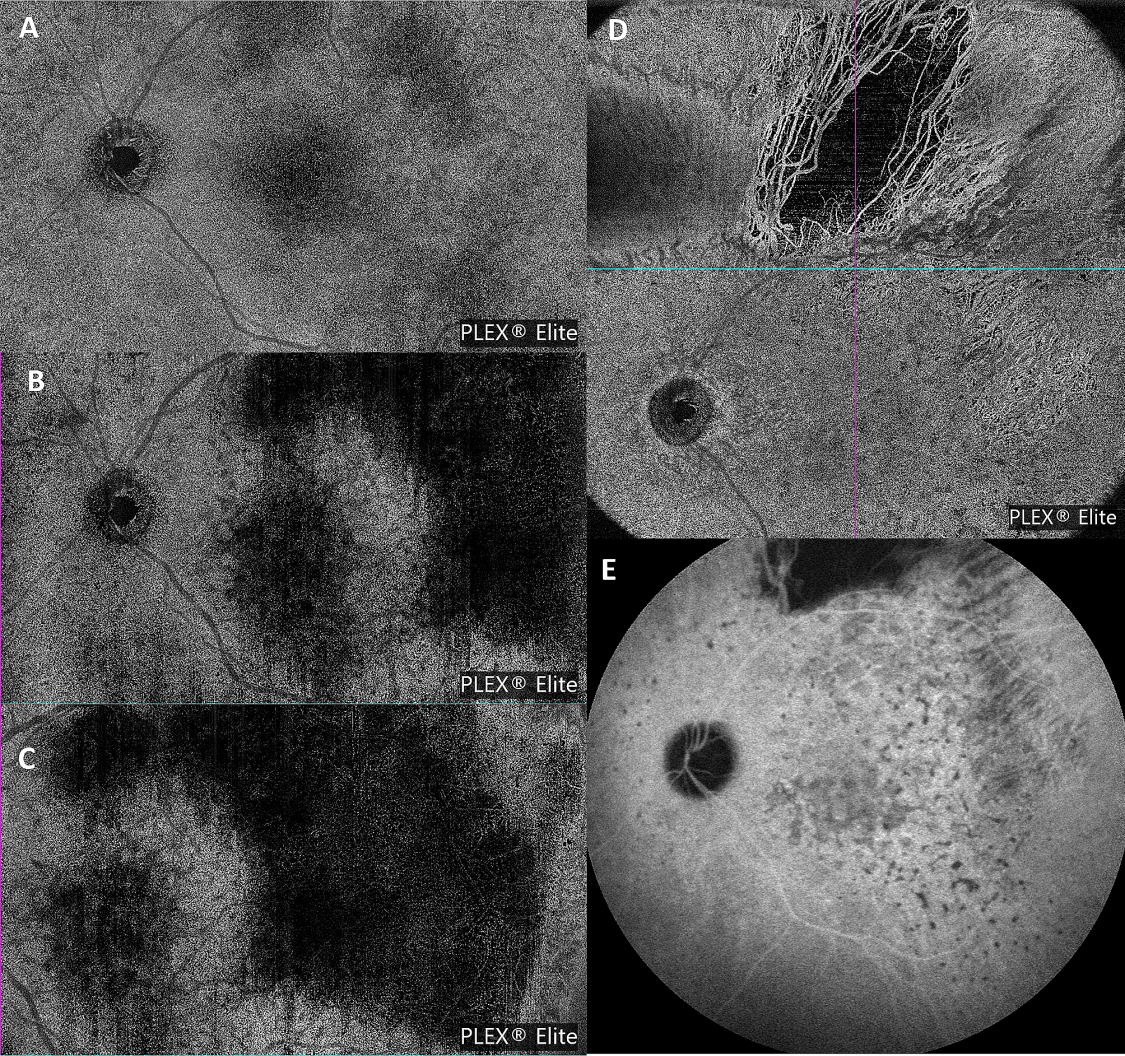
(**A**) SS-OCT-A choriocapillary analysis at baseline, revealing circumscribed low reflectance areas in foveal and parafoveal areas. (**B**, **C**) SS-OCT-A choriocapillary analysis performed seven weeks after the presentation, showing an extension of the low reflectance areas in the central posterior pole and superior temporal quadrant. (**D**) SS-OCT-A choriocapillary analysis after chemotherapy and autologous stem cell transplant, showing a remarkable reduction of the large central and superior temporal low reflectance areas, with the persistence of some hyporeflective central spots. Similar changes were detected on ICGA examination (**E**)

Three weeks later, an increase in sub-RPE deposits in the LE was detected on OCT, and the presence of a large flat pigment epithelial detachment (PED) in the superotemporal quadrant was observed, along with an increase in the thickness and reflectivity of overlying retina (Fig. 4A, B,C). In this area, the PFBLs were more numerous and appeared in continuity with the thick and hyperreflective overlying retina (Fig. 4C). On SS-OCT-A, a choriocapillary analysis showed an extension of the low reflectance areas, which affected the central posterior pole and superotemporal quadrant (Fig. 5B, C). In the RE, yellowish white lesions in the upper part of the posterior pole were detected upon fundus examination, which appeared as sub-RPE hyper-reflective deposits on OCT, and which were hyperautofluorescent on AF.

A chorioretinal biopsy was performed, collecting a specimen from the thickened tissue in the superior temporal quadrant of the posterior pole. The specimen was put in formaldehyde and sent to a National Referral Center for histological analysis. A dense, large lymphoid population was found, which was immunoreactive for CD20, bcl-2, and MUM-1, partly so for bcl-6, and negative for CD3m and CD10.The immunocytochemical determination of Ki67 (clone 30 − 9) performed on a histological section in paraffin demonstrated about 80–90%. A diagnosis of a large B-cell lymphoma with non-germinal centre B cell-like phenotype was formulated. The patient underwent cancer staging, including a bone marrow biopsy and a Positron Emission Tomography, which showed no other disease foci.

In the meantime, LE visual acuity had decreased to 20/100, while still 20/20 in the RE. Multimodal imaging showed spreading in the mid-periphery of the sub-RPE lesions in the RE, in the same fashion previously seen in the LE.

For the treatment, the patient was referred to a national reference center for Oncological Hematology. He underwent three chemotherapy cycles, followed by autologous bone marrow stem cell sampling, two additional chemotherapy cycles, and a final stem cell reimplantation. Eight months after the chorioretinal biopsy and 2 months after completing chemotherapy and the autologous stem cell transplant, LE visual acuity increased to 20/20.

**Fig. 6:**
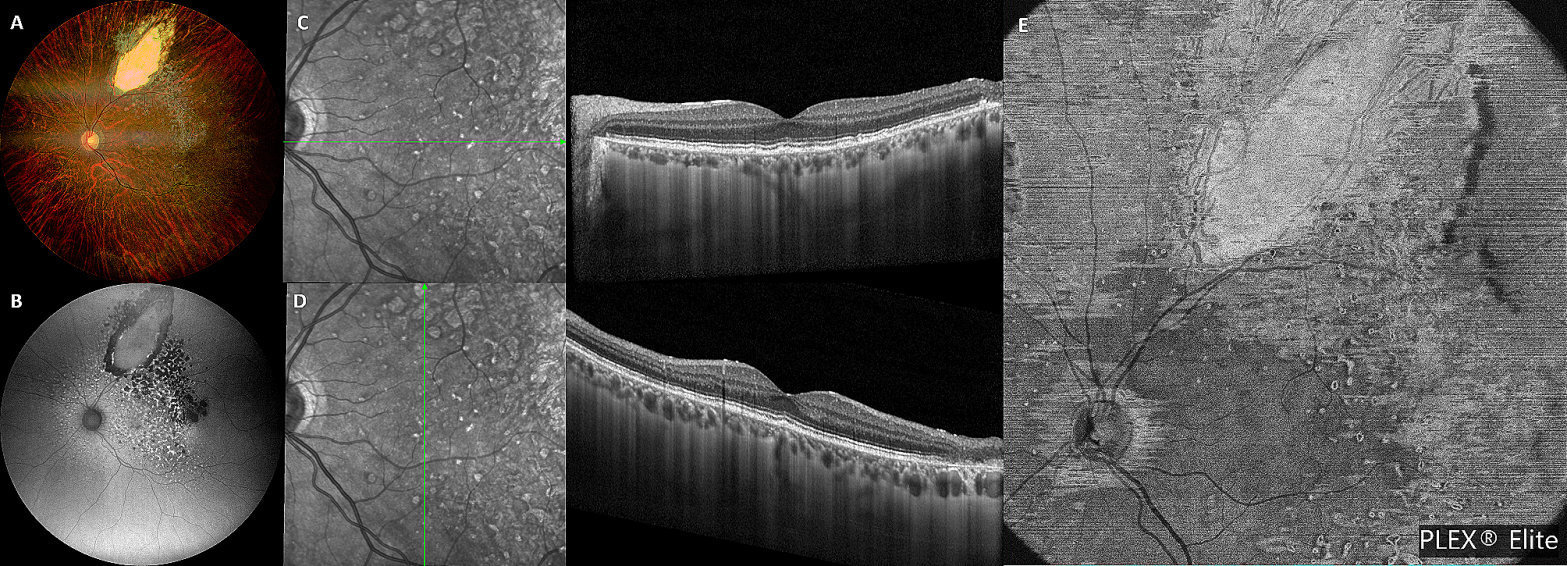
Multimodal imaging after chemotherapy and autologous stem cell transplant. (**A**) Color fundus photo showing sharp scar margins at the chorioretinal specimen collection site. (**B**) Autofluorescence showing a reduction in hyperautofluorescen lesions. (**C**, **D**) OCT scans performed 10 months after the first presentation (corresponding to eight months after chorioretinal biopsy, and 2 months after completing chemotherapy and autologous stem cell transplant) showing remarkable reduction of sub-RPE deposits and SND disappearance. (**E**) SS-OCT-A enface outer retina slabs showing reduction of the multiple hyperreflective alterations, corresponding to the sub-RPE deposits

**Fig. 7:**
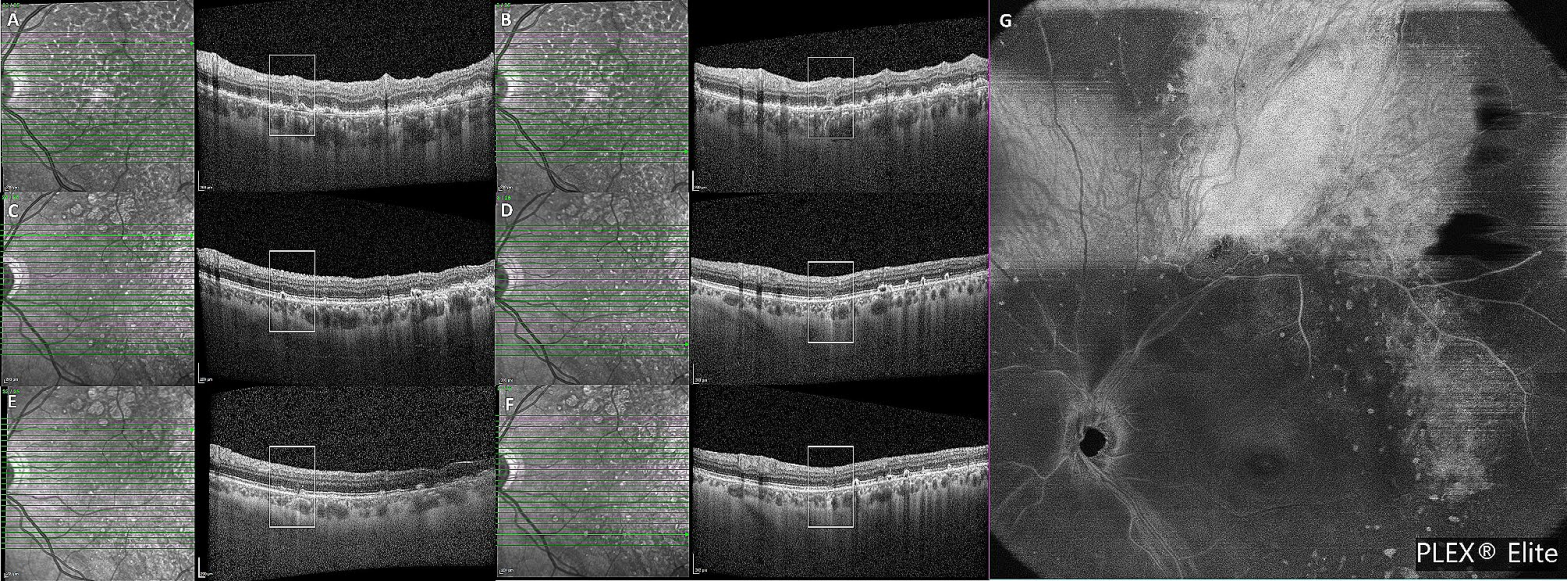
Evolution of the PFBLs over 3 years. (**A**, **B**) One month after the first presentation. OCT scans showing PFBLs as hyperreflective vertical-shaped retinal lesions. (**C**, **D**) After 1 year. OCT scans showing PFBLs regression, alongside dystrophic/atrophic RPE alteration onset their sites. (**E**, **F**) After 2 years. Dystrophic/atrophic RPE alterations detectable on OCT at the PFBL location. (**G**) SS-OCT-A at 1 year after presentation. Regression of the hyperreflective spots on enface midretinal slabs, which corresponded with the PFBLs

The patient was then followed-up in our department for three years. Multimodal imaging examination performed during follow up visits showed changing retinal lesions. On OCT examination, sub-RPE deposits had reduced remarkably, SND had disappeared (Fig. 6C, D), and the superior temporal flat PED had regressed along with the thickening of the overlying retina (Fig. 4D). On SS-OCT-A, a reduction in the multiple hyperreflective alterations corresponding to the sub-RPE deposits was detected on enface outer retina slabs (Fig. 6E). The PFBLs had regressed, and in their sites dystrophic/atrophic RPE alterations were detected on OCT (Fig. 7 shows evolution of the PFBLs over 3 years). SS-OCT-A examination showed regression of the hyperreflective spots on the enface midretinal slabs that had corresponded to the PFBLs (Fig. 7G).

On SS-OCT-A analysis, choriocapillary reconstruction showed a remarkable reduction of the large low reflectance areas, with the persistence of some hyporeflective central spots (Fig. 5D). Similar changes were detected on ICGA examination (Fig. 5E).

At the chorioretinal specimen collection site, the scar margins were sharp, and no signs of proliferative vitreoretinopathy (PVR) were present on fundus examination. At the last FU visit, a posterior polar cataract was found in the LE. Follow-up PET and MRI were negative.

## Discussion and conclusions

VRL diagnosis can be challenging due to the wide range of clinical presentations, including similarities with chronic uveitis. Moreover, when a VRL diagnosis is clinically suspected, reaching confirmation can be problematic as well [8]. In our case, a young man with no relevant medical or ocular history was diagnosed with VRL after a challenging assessment path, where many procedures turned out to be negative, making the diagnostic confirmation even more difficult. Multimodal imaging improved the diagnostic suspicion, guided the ensuing diagnostic procedures, and showed treatment response. When compared with previous literature, novel features were identified on SS-OCT-A and uncommonly described findings were confirmed.

Diagnostic vitrectomy was performed as the first procedure to confirm VRL. The cytological analysis of vitreous biopsies obtained during PPV remains a hallmark procedure in diagnosing the disease. However, its sensitivity varies widely from 31 to 87.5% [9, 10]. Many factors can affect the result, including the cellularity of the sample and the expertise of the pathology labs [9, 10]. Lymphoma cells may undergo morphological degradation within 60 min in absence of appropriate preservatives and when samples are not gently handled. Moreover, the cellularity may be reduced by corticosteroid treatments. In our case, the negative result could also be explained by the absence of any vitreous involvement. International consensus recommendations suggest that diagnostic vitrectomy is preferred in cases with vitreous involvement [9].

IL-10:IL-6 ratio analysis on a vitreous sample was also considered in our case. IL-10 levels tend to be elevated in the presence of malignant B-lymphocytes, whereas IL-6 is elevated in inflammatory states. Therefore, an IL-10:IL-6 ratio of > 1 is considered in favor of a B-cell lymphoma. However, the use of the IL-10:IL-6 ratio is controversial. In fact, elevation may occur in eyes with non-neoplastic uveitis. In one report, the IL-10:IL-6 ratio was elevated in 8 out of 14 vitreous samples with non-neoplastic vitritis. An IL-10:IL-6 ratio < 1 has been found in known cases of VRL [10]. Moreover, it has also been reported that in the presence of extensive and severe sub-RPE or retinal infiltration, as we found in our case, the IL-10:IL-6 ratio may not be typical and should thus be interpreted carefully [9]. The IL10:IL6 ratio was not performed in our case because it was not available. Nevertheless, for the reasons mentioned, it would not have been enough to achieve a definitive conclusion.

In a similar vein, MYD88 mutation analysis may be considered a valuable additional tool in VRL diagnosis [9], but a negative result may not definitively rule out the diagnosis since MYD88 mutations have been found in only up to 70% of all VRL [11].

Given the negative result of the above procedures, a chorioretinal biopsy was performed for our case, which confirmed the clinically suspected diagnosis. This approach has been helpful in other cases where previous diagnostic procedures proved inconclusive [12, 13].

Considering the invasive nature of the procedures for a VRL diagnosis, better knowledge of multimodal imaging features of the disease would be desirable to increase pre-test probability. Previous studies described several VRL features [5, 14, 15]. However, few studies have reported SS-OCT-A findings, and their long-term follow-up remains unexplored. In this report, we describe various retinal findings in a case of VRL over a three-year FU, focusing on SS-OCT-A features. Our analysis led to the detection of novel SS-OCT-A features and confirmed uncommonly described findings that were analyzed throughout the three-year FU.

Outer-retinal abnormalities in VRL patients have been described in several previous studies. They are thought to be due to the infiltration of malignant lymphoma cells into the retina. Sub-RPE deposits, also found in our case, have mainly been examined with OCT [5, 8, 14] and AF [15] Pierro et al. [7] firstly described their appearance on SS-OCT-A as multiple hyperreflective spots on enface outer retina slabs and SS-OCT-A analysis, thus interpretable as a movement signal provided by white cell infiltration. Similarly, in our case, sub-RPE deposits appeared hyperreflective on enface SS-OCT-A outer retina slabs (Fig. 1F). We also analyzed this feature during the FU, finding a remarkable reduction of the hyperreflective lesions previously detected on SS-OCT-A (Fig. 6E) alongside the reduction of sub-RPE deposits on OCT.

Recently, Chen et al. [6] described PFBLs as novel characteristics of VRL on OCT-A, highlighting their possible role in facilitating early diagnosis and appearing as vertical intraretinal lesions. These features were also found in our case (Fig. 3). They were absent upon initial presentation, only appearing one month later during the diagnostic confirmation process and thus interpretable as a sign of disease progression. Moreover, we found that three weeks later, the PFBLs had become more numerous alongside an increase in sub-REP infiltration, and in some areas, they were in continuity with alterations in the overlying retina which appeared thick and hyperreflective. This may be interpretable as further disease progression with more extensive infiltration of malignant lymphoma cells into the retina. In the ensuing FU, we found a regression of VRL both on enface SS-OCT-A and on OCT scans in which they were replaced by dystrophic/atrophic RPE alteration. In parallel, a regression of the other sign of infiltration was detected.

Unlike Pierro et al. and Chen et al., we also found alterations in SS-OCT-A choriocapillary analysis. It firstly revealed circumscribed low reflectance areas in foveal and parafoveal areas that subsequently evolved into larger hyporeflective areas, which affected the central posterior pole and superior temporal quadrant (Fig. 5). Notably, these areas were also those most affected by retinal thickening and hyperreflectivity on OCT. They reduced considerably after treatment. We could not find any description of these SS-OCT-A findings in any previous study, since the choriocapillary plexus reconstruction has always been described as revealing no alteration. The clinical meaning of such alterations is yet to be understood. They could be interpretable as areas of flow signal impairment. However, they are more likely due to a shadowing artifact from the overlying infiltrated retinal tissues.

In conclusion, greater knowledge of VRL features would be helpful for timely diagnosis and therapy. In our case, we report multimodal imaging findings and long-term FU in a case of VRL, focusing on SS-OCT-A features. We describe novel SS-OCT-A findings and the evolution of uncommonly reported features, adding to current knowledge on the clinical presentation of VRL. Moreover, these aspects could provide avenues for future study on the characteristic presentation of the disease and improve disease understanding.

## Data Availability

All data and material are included in the manuscript and the figures.

## Abbreviations

VRL: vitreoretinal lymphoma
PCNSL: primary central nervous system lymphoma
CNS: central nervous system
OCT: optical coherence tomography
AF: autofluorescence
FA: fluorescein angiography
ICGA: indocyanine green angiography
SS-OCT-A: swept-source OCT angiography
BCVA: best-corrected visual acuity
LE: left eye
RE: right eye
SND: subfoveal neuroretinal detachment
RPE: retinal pigment epithelium
PED: pigment epithelial detachment
PPV: pars plana vitrectomy
IL-10: interleukin 10
IL-6: interleukin 6
PVR: proliferative vitreoretinopathy
FU: follow-up
